# Co-Channel Interference Suppression for LTE Passive Radar Based on Spatial Feature Cognition

**DOI:** 10.3390/s22010117

**Published:** 2021-12-24

**Authors:** Haitao Wang, Xiaoyong Lyu, Kefei Liao

**Affiliations:** 1 School of Information and Communication, Guilin University of Electronic Technology, Guilin 541004, China; wanghaitao@mails.guet.edu.cn (H.W.); kefeiliao@guet.edu.cn (K.L.); 2 School of Information Engineering, Zhengzhou University, Zhengzhou 450001, China

**Keywords:** passive radar, long term evolution (LTE) signals, co-channel interference suppression, spatial feature cognition

## Abstract

Passive radars based on long-term evolution (LTE) signals suffer from sever interferences. The interferences are not only from the base station used as the illuminator of opportunity (BS-IoO), but also from the other co-channel base stations (CCBS) working at the same frequency with the BS-IoO. Because the reference signals of the co-channel interferences are difficult to obtain, cancellation performance degrades seriously when traditional interference suppression methods are applied in LTE-based passive radar. This paper proposes a cascaded cancellation method based on the spatial spectrum cognition of interference. It consists of several cancellation loops. In each loop, the spatial spectrum of strong interferences is first recognized by using the cyclostationary characteristic of LTE signal and the compressed sensing technique. A clean reference signal of each interference is then reconstructed according to the spatial spectrum previously obtained. With the reference signal, the interferences are cancelled. At the end of each loop, the energy of the interference residual is estimated. If the interference residual is still strong, then the cancellation loop continues; otherwise it terminates. The proposed method can get good cancellation performance with a small-sized antenna array. Theoretical and simulation results demonstrate the effectiveness of the proposed method.

## 1. Introduction

Passive radar uses the third-party emitters as the illuminators of opportunity (IoO) for target detection and tracking [1,2]. This technique has a number of advantages. For example, passive radar imposes no interference to the electromagnetic environment and it does not require spectrum allocation; therefore, a passive radar network is easier to form for improved detection performance. Additionally, the operation of passive radar is covert and the cost is controllable. The available third-party irradiation sources used as the IoO of passive radar mainly include frequency modulation (FM) broadcast signal [3,4], digital audio/television broadcast (DAB/DVB) [4,5,6], global navigation satellite system (GNSS) [7], long term evolution (LTE) signal [8,9,10], and so on. Specifically, the LTE signal when used as the IoO of passive radar has many advantages: (1) the LTE base stations are densely distributed; multiple stations can be selected easily to form a multi-static radar that can provide great spatial diversity for improved detection capability. (2) The LTE standard adopts the orthogonal frequency division multiplexing (OFDM) and multiple input multiple output (MIMO) technology and flexible bandwidth configuration to realize high-rate data transmission. Its maximum bandwidth is 20 MHz, which provides good range resolution for radar use. These characteristics encourage the utilization of the LTE for passive radar [8].

Usually, target echo in passive radar is contaminated by the direct and multipath interference (DMI) signals [11], and it is far weaker than DMI. Therefore, interference cancellation should first be performed to suppress the strong interference and increase the target signal to interference ratio. Many time-domain and spatial filtering algorithms have been developed to suppress these interference signals, such as the least mean square (LMS) [2,12], extensive cancellation algorithm (ECA) [13], and subcarrier domain algorithm [14,15]. These methods usually first collect the direct signal by using a narrow-beam antenna directed to the transmitter and then estimate the amplitude and delay of each interference signal. Finally, the interference signals are subtracted from the surveillance signals with its estimated parameters.

In traditional passive radars, such as the passive radars based on FM and analog TV signals, the transmission adopts the frequency division multiple access (FDMA) technique and transmitters working on the same frequency are far away from each other. Therefore, the interference signals mainly include the DMI from the transmitter that is used as the IoO (T-IoO) [16]. The co-channel interference is usually ignored. However, the interference signals in LTE passive radar not only include the DMI from the base station used as the IoO (BS-IoO), but also the DMI from the co-channel base stations (CCBS) that work at the same frequency channel as the BS-IoO [10,17]. This is because the base stations in 4G-LTE mobile communication systems are densely distributed and some of them share the same frequency band owing to the frequency re-use scheme in 4G-LTE. We name the DMI from the CCBS as the co-channel interference hereafter. In passive radar, the target echo is quite weak and the co-channel signals can be far stronger than the target echo, which causes severe interference on the target detection [18,19]. The co-channel interference also arises in DTV passive radar, where the interference has the same waveform as the DMI from the T-IoO [20]. The traditional cancellation methods have been customized to suppress both the DMI and the co-channel interference in DTV passive radar. However, the co-channel interference in LTE passive radar usually has different waveforms with the DMI of the BS-IoO, therefore the traditional cancellation methods cannot be used to suppress this interference, and a new method has to be developed. 

In order to suppress the co-channel interference, the reference signal of each interference source should be obtained. The clean reference signal can be found by directing a narrow beam in the direction of the specific CCBS, which is unfortunately not known in advance usually. The directions of the CCBSs can be obtained by estimating the directions of the corresponding arriving signals. Because the LTE base stations are densely distributed, the traditional MUSIC and CAPON spatial spectrum estimation algorithms cannot get an accurate estimation of the directions of the arriving signals because the number of signals far exceeds that of the array elements. In this paper we propose a cascaded cancellation method for suppressing the DMI and co-channel interference in LTE passive radar. We first propose a spatial spectrum recognition method based on the cyclostationary characteristics of the LTE signal and the compressed sensing technique, through which we can estimate the spatial distribution of the interference signals. The reference signal of each strong interference base station is then obtained through beamforming, and a clean version of the reference signal is reconstructed with the signal bits demodulated from the received signal. Third, the co-channel interference from each base station is cancelled. Finally, a termination criterion is checked to determine whether the interference in the remaining signal has been adequately suppressed. If not, the cancellation procedure above is repeated. The termination of the cancellation procedure is determined by comparing the interference to noise ratio (INR) in the remaining signal with a predefined threshold. The INR is estimated through a cycle autocorrelation method and is referred to in our previous work [10]. If the estimated INR is smaller than the threshold, then the cascaded cancellation algorithm terminates. The advantages of the proposed method are two-fold. First, the proposed method can obtain a good estimation of the spatial distribution of the strong interference signals even with a small sized antenna array. Additionally, the proposed method uses a termination criterion to check whether the interference is suppressed adequately. If not, the cancellation procedure repeats. In contrast, the traditional cancellation methods only perform once and there is no indicator about how the interference is suppressed. The proposed method is analyzed through a range of simulations. The results demonstrate the effectiveness of the proposed method. 

## 2. Signal Model

**Figure 1:**
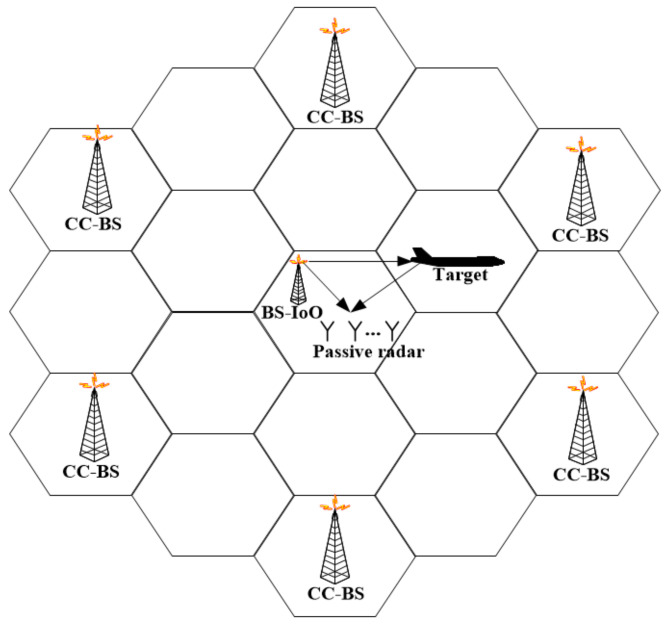
Schematic diagram of radar target detection structure for LTE passive radar.

Consider the LTE passive radar shown in Figure 1. A linear antenna array with M elements is exploited. The spacing interval between the adjacent elements is one half of the wavelength. For this antenna array, the range of the unambiguous angle measurements is from −90° to 90°. The received signal of each antenna array element can be represented as:

(1) $$s_{m}[n]=t_{{\mathrm{tar}}_{m}}\left[n\right]+z_{m}[n]+I_{m}[n]\;\;n=1,\ldots ,N\;m=1,\ldots M$$

where

(2) $$t_{{\mathrm{tar}}_{m}}[n]=d_{1}[n-\tau_{ar}]e^{j\pi m\sin(\theta_{tar})}\;$$

(3) $$I_{m}[n]=\sum_{s=1}^{N_{s}}\sum_{i=1}^{N_{I}{}^{s}}a_{s,\;i}d_{s}[n-\tau_{s,\;i}]e^{j\pi m\sin(\theta_{s,i})}\;n=1,\ldots ,N\;m=1,\ldots M$$

where $t_{{\mathrm{tar}}_{m}}[n]\;$ is the target echo; $I_{m}[n]$ is the co-channel interference signal from the CCBSs;$z_{m}[n]$ is thermal noise; N is the total number of the signal samples; $d_{1}[n]$ is the reference signal of the BS-IoO; $\tau_{ar}$ is the delay of a specific target; $\theta_{tar}$ is the target’s direction of arrival (DOA); $d_{s}[n]$ is the direct signal from the *s*-th CCBS, where we assume the base station 1 is the BS-IoO and 2-Ns are the CCBSs; $\tau_{s,\;i}$, $a_{s,\;i,m}$ and $\theta_{s,\;i}$ are the delay, complex amplitude, and DOA of the *i*-th multipath signal from the s-th CCBS; $N_{I}{}^{s}$ is the number of the multipath signals from the *s*-th CCBS; and $N_{s}$ is the number of the CCBSs within the field of surveillance.

As can be seen from Equation (2), interference signals in LTE passive radar not only include the DMI from the BS-IoO, but also the co-channel interference signals from the CCBSs. This is different from traditional passive radars such as the FM and analog TV passive radars, where only the DMI from the BS-IoO is considered. Usually, the waveforms of the co-channel interference signals from different CCBSs are different, and they are different from that of the direct signal of the BS-IoO. Therefore, it is not possible to suppress the co-channel interference signals with the direct signal of the BS-IoO. The direct signal of each interference should be collected. A straightforward way to collect the direct signal of an interference base station is by forming a narrow beam to the direction of the transmitter. In this circumstance, the direction of arrival of the interference base station should be known. In the following, we will introduce a spatial spectrum cognition method to estimate the spatial distribution of the interference signal sources. We then use the beamforming technique to form a narrow beam to the direction of the interference base station and get the direct signal. A clean version of the direct signal is further reconstructed with the signal bits demodulated from the received signal. With the direct signal, each interference signal is cancelled and the remaining interference signal to noise ratio is estimated through a cyclic autocorrelation method to assess the inference level. If the INR is smaller than a predefined threshold, then the cancellation terminates. Otherwise the cancellation procedure repeats.

## 3. Algorithm Description

**Figure 2:**
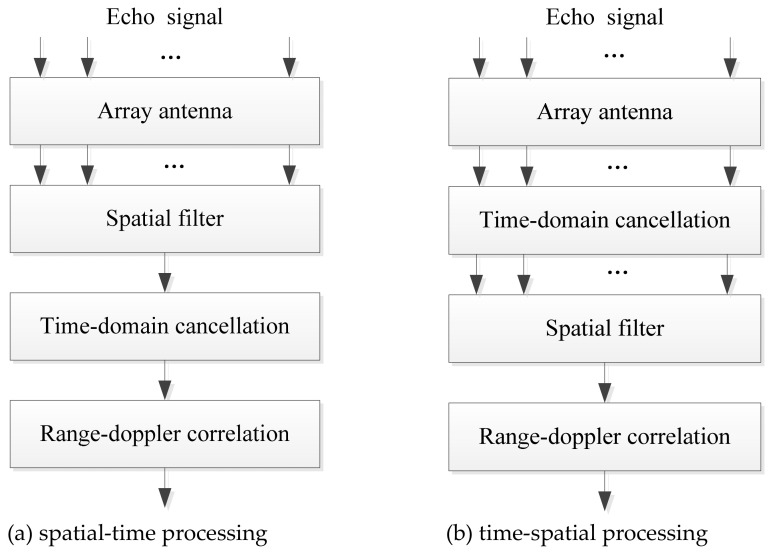
Flow charts of the (**a**) spatial-time and (**b**) time-spatial processing.

Traditional passive radar interference suppression methods include the time-domain and spatial-domain cancellation methods. To achieve the best effect of interference suppression, joint space-time cascaded processing is usually adopted [21,22,23,24]. According to the order of the time domain processing and spatial domain processing, the joint space-time cascaded processing can be divided into spatial-time processing [21,22] and time-spatial processing [23,24]. The processing flow charts of the two methods are shown in Figure 2.

The spatial-time processing first performs spatial filtering to suppress the interference signals and then conducts the time-domain adaptive filtering to further suppress the DMI of the BS-IoO. In contrast, the time-spatial processing first conducts time-domain adaptive filtering to cancel the DMI of the BS-IoO and then performs spatial filtering to further suppress the interference residual. The traditional methods above only consider the suppression of DMI from the BS-IoO but never consider the suppression of co-channel interferences. That is, the time-domain cancellation in their processing flow charts only uses the reference signal of the BS-IoO to conduct the cancellation. It cannot suppress the co-channel interference signals, as the co-channel interference signals have different waveforms from the reference signal of the BS-IoO. The spatial filtering not designated to cancel the co-channel interference may suppress the co-channel interference to some extent. However, the CCBSs in LTE communication systems are densely distributed and the number of interference signal sources are usually very large. In order to get adequate suppression for traditional spatial filtering, the number of antenna array elements required must be larger than that of the interference signal sources, which will lead to over-large antenna size. 

**Figure 3:**
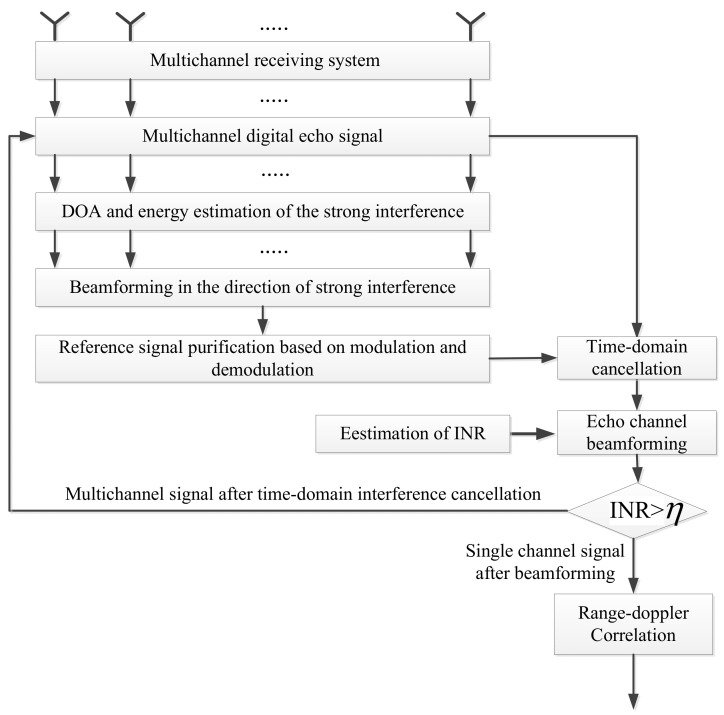
The proposed co-channel interference cascaded cancellation algorithm.

In the following, we propose a cascaded cancellation method based on the spatial feature cognition of the interference signals, which can get adequate cancellation performance with a small sized antenna array. The processing flow chart of the proposed method is shown in Figure 3. 

It can be seen from the figure that the proposed algorithm consists of several cancellation loops. Each loop mainly includes the following processing steps:

(1) DOA and energy estimation of the strong interference:

In practice, the DOA of the BS-IoO is usually known a priori. However, it is not easy to know the positions of all the CCBSs. Therefore, we propose to estimate the DOAs of the interference signals from the CCBSs by processing the received surveillance signal. We utilize the cyclostationary characteristics of LTE signals and the compressed sensing technique to get a good DOA estimation performance with only a small sized antenna array. In this step, we also estimate the energy of the interference signals.

(2) Beamforming in the direction of strong interference:

With the DOA of the strong interference signals, we use the beamforming technique based on the worst-case performance optimization [25] to generate a beam directing to each strong interference. This beamforming technique can generate a beam directing to the desired interference direction; it can also suppress the interference signals from the other directions, so as to maximize the signal-to-noise ratio. At this step, we can get the direct signal of each interference base station.

(3) Purification of the direct signal based on demodulation and reconstruction:

The direct signal of each interference base station obtained by using the beamforming technique above can inevitably be contaminated by the other interference residual. Here, we demodulate the direct signal to get the signal bits and then reconstruct the direct signal according to the LTE protocol [26] to get the clean direct signal. At this step, the clean direct signal from each interference base station can be obtained and it is used as the reference signal by the time-domain cancellation method to suppress the corresponding interference signal.

(4) Time-domain interference cancellation:

Based on the reference signals obtained in the previous step, the ECA-B algorithm proposed in [13] is used to eliminate the interference signals from each CCBS.

(5) Spatial beamforming in the surveillance region:

The beamforming technique based on the worst-case performance optimization is exploited again to generate a beam directing to the surveillance region of interest to resolve the target echo. At the same time, this operation can further suppress the interference residual.

(6) Evaluation of interference suppression effect:

In this step, we use the CAC method proposed in our previous work [10] to estimate the interference to signal noise (INR) in the remaining signal. The INR is used to evaluate the level of interference residual. If the interference residual is still strong, the cascaded cancellation loop continues to further suppress the interference.

It can be seen from the preceding cancellation procedure, the core step is the direction finding of the interference at each cancellation loop. In the following, we explain the direction-finding method in more detail.

### 3.1. Direction Finding of the Interference based on the Cyclostationary Characteristics of the OFDM Signal and the Sparse Reconstruction Technique

**Figure 4:**
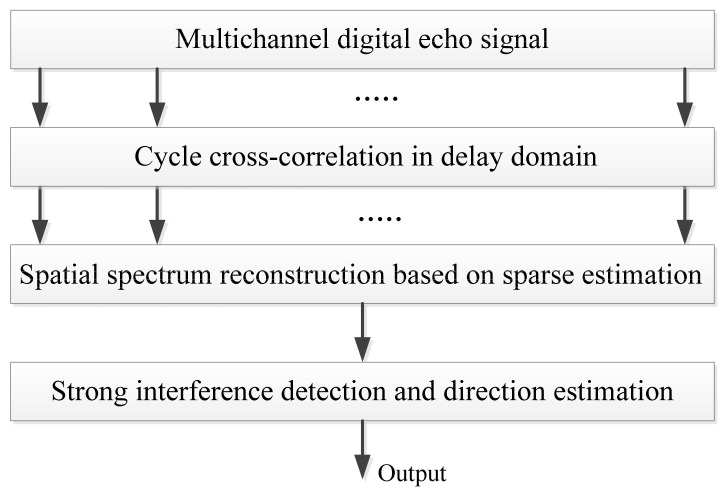
Strong disturbance direction and energy estimation based on the OFDM cyclostationary characteristics and sparse reconstruction.

It can be seen from the signal model described by Equation (1) that the surveillance signal consists of many co-channel interference signals. It is not easy to use the traditional spatial spectrum estimation method to estimate the DOAs of the signal sources. In this section we propose a method that utilizes the cyclostationary characteristics of the OFDM signal and the sparse reconstruction technique to estimate the DOAs of the interference signals. By doing so, only a small sized antenna array is necessary to get an adequate DOA estimation. The flow-chart of the estimation algorithm is shown in Figure 4.

#### 3.1.1. Cycle Cross-Correlation (CCC) in Delay Domain

First, the cycle cross-correlation between the signal received by the first antenna array element and the signals from the other elements is performed to improve the SNR of the signal.

(4) $$y_{m}[k]=\sum_{n=1}^{N}s_{1}\left(n\right)s_{m}{}^{*}\left(n-k\right)\;k=1,\ldots ,K,\;m=1,\ldots M$$

It can be assumed that the interference signals from different CCBSs, target echo, and noise are statistically independent. Therefore, the cross-correlation products between different CCBSs, target echo, and noise can be omitted. Equation (4) can then be approximated as:

(5) $$y_{m}[k]\approx \sum_{n=1}^{N}I_{m}\left(n\right)I_{1}{}^{*}\left(n-k\right)+\sum_{n=1}^{N}z\left(n\right)z_{1}{}^{*}\left(n-k\right)+\sum_{n=1}^{N}t_{{\mathrm{tar}}_{m}}\left(n\right)t_{{\mathrm{tar}}_{1}}{}^{*}\left(n-k\right)$$

Because the target echo is far weaker than the interference signals, it can be ignored when estimating the directions of the interference signals. Equation (5) can be further simplified as:

(6) $$y_{m}[k]\approx \sum_{n=1}^{N}I_{m}\left(n\right)I_{1}{}^{*}\left(n-k\right)+\sum_{n=1}^{N}z_{m}\left(n\right)z_{1}{}^{*}\left(n-k\right)$$

**Figure 5:**
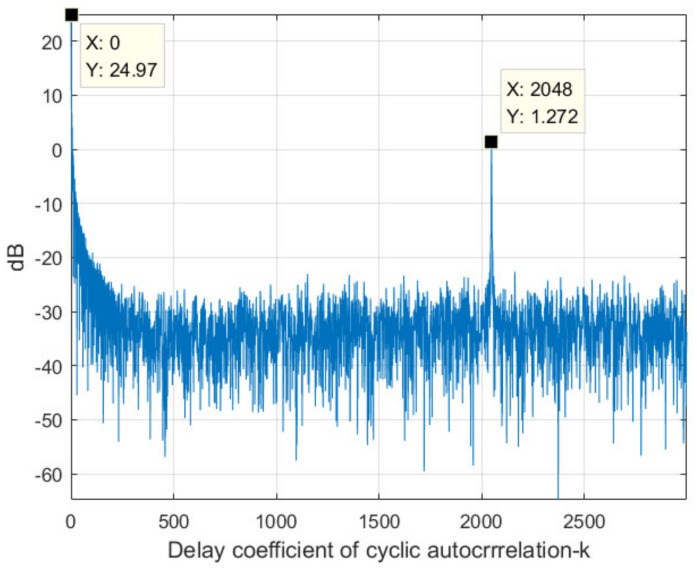
CAC result of a simulated LTE signal.

Figure 5 shows the cross-correlation result of the signals received by two different antenna array elements. The signals are simulated according to the LTE signal protocol, where the sampling rate is set to 30.72 MHz, i.e., the code rate of the LTE signal. It can be seen from the figure that there are two peaks at *k* = 0 and *k* = 2048.

In the following, we will analyze the reasons why the two peaks arise and their influences on the estimation of the interference signal direction.

For *k* = 0, Equation (6) can be rewritten as:

(7) $$\begin{array}{l} y_{m}[0]\approx \sum_{n=1}^{N}I_{m}\left(n\right)I_{1}{}^{*}\left(n\right)+\sum_{n=1}^{N}z_{m}\left(n\right)z_{1}{}^{*}\left(n\right)\\ \;\;\;=\sum_{s=1}^{N_{s}}\sum_{i=1}^{N_{I}{}^{s}}A_{s,\;i}P_{s,i,m}+\sum_{n=1}^{N}z_{m}\left(n\right)z_{1}{}^{*}\left(n\right) \end{array}$$

where $A_{s,\;i}=a_{s,\;i}{}^{2}\sum_{n=1}^{N}d_{s}[n-\tau_{s,\;i}]d^{*}{}_{s}[n-\tau_{s,\;i}]$ represents the energy of the interference signal after CCC. We can get the same term when performing the cross-correlation of the signals between any two array element channels. $P_{s,i,m}=e^{j\pi m\sin(\theta_{s,i})}e^{-j\pi \sin(\theta_{s,i})}=e^{j\pi (m-1)\sin(\theta_{s,i})}$ is the phase function of the cross-correlation result. It can be seen that, for k = 0, $A_{s,\;i}$ represents the autocorrelation of the interference signal. After CCC, the energy of the interference signal is integrated to a large magnitude.

The expression $\sum_{n=1}^{N}z_{m}\left(n\right)z_{1}{}^{*}\left(n\right)$ is the cross-correlation result corresponding to the noise. For *m* = 1, it represents the autocorrelation of the noise from channel 1. For *m* = 2,…, *M*, it represents the cross correlation of the noise from different channels, which is statistically independent and cannot be integrated.

As can be seen from the above analysis, when *k* = 0, all the interference signals can achieve a complete match, so the highest peak as shown in Figure 5 can be obtained. At the same time, when *m* = 1, the noise can also achieve a complete match. Thus the INR will not increase for *m* = 1. However, when *m* = 2, 3,…, *M*, the noise energy cannot be accumulated due to the statistical independence of noises in different array element channels. Therefore, for *m* = 2, 3,…, the INR of each interference signal after CCC can be increased. Therefore, after CCC, the INR for *m* = 1 is inconsistent with that for *m* = 2, 3,…, *M*. Therefore, If DOA and energy are estimated using the cross-correlating result at *k* = 0, amplitude modulation exists, which will affect the estimation accuracy.

For *k* = 2048, Equation (6) can be rewritten as:

(8) $$\begin{array}{l} y_{m}[2048]\approx \sum_{n=1}^{N}I_{m}\left(n\right)I_{1}{}^{*}\left(n-2048\right)+\sum_{n=1}^{N}z_{m}\left(n\right)z_{1}{}^{*}\left(n-2048\right)\\ \;\;\;=\sum_{s=1}^{N_{s}}\sum_{i=1}^{N_{I,}{}^{s}}B_{s,\;i}P_{s,i,m}+\sum_{n=1}^{N}z_{m}\left(n\right)z_{1}{}^{*}\left(n-2048\right) \end{array}$$

where $B_{s,\;i}=a_{s,\;i}{}^{2}\sum_{n=1}^{N}d_{s}[n-\tau_{s,\;i}]d^{*}{}_{s}[n-\tau_{s,\;i}-2048]$ represents the CAC of the interference signal with one of the signals delayed by 2048 delay bins.

**Figure 6:**
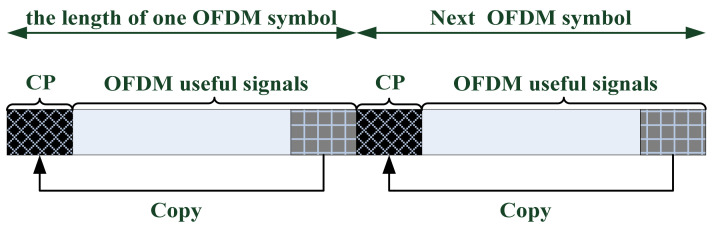
LTE signal OFDM modulation schematic diagram.

In the following we analyze $B_{s,\;i}$ in details. According to the protocol specification of 3GPP, the LTE systems adopt OFDM modulation, and its modulation structure is shown in Figure 6.

**Figure 7:**
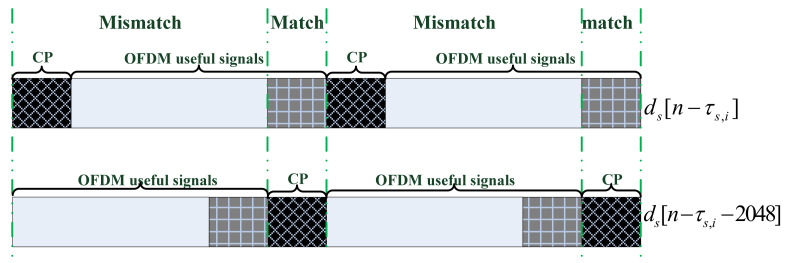
Cyclic autocorrelation of the LTE signal.

As can be seen from the figure, the OFDM signal consists of multiple symbols in the time domain. The data content of each symbol includes two parts, namely, the useful signal part and the cyclic prefix (CP) part. The CP is mainly used to combat intersymbol and multipath interference and it is a copy of the last segment of the useful signal (as shown in the figure above). Therefore, the LTE signal has the characteristics of cyclostationarity, and the cyclostationarity period is 2048, i.e., the length of the OFDM useful signal. Figure 7 shows schematically the cyclic autocorrelation of the LTE signal with its delay replica, of which the delay is 2048 bins:

It can be seen from the above figure that when performing the CAC with one of the signals delayed by 2048 points, the CP of the two signals match. The integrated gain of the CAC is proportional to 144×D, where 144 is the length of CP in each OFDM symbols and D is the number of symbols contained in the LTE signal. Because noise has no cyclostationarity, it cannot integrate in the CAC results. Therefore, the INRs for *m* = 1, 2,…, *M* are increased to the same amount, which is different from the case described in Equation (7).

It can be seen from the above analysis that when *k* = 0, although the INRs for *m* = 2, 3,…, *M* can be improved, the INR for *m* = 1 will not be improved. Therefore, the cyclic cross-correlation result at *k* = 0 will have amplitude modulation for different antenna array element channels, which will have a great impact on the direction estimation accuracy. When *k* = 2048, the INR for each channel can be increased by the same amount. In addition, the other co-frequency and adjacent frequency interference signals that are not LTE signals will also be suppressed when *k* = 2048, as they have no cyclostationarity. This means that the impact of non LTE interference on DOA estimation of the LTE co-channnel interference can be eliminated. Therefore, the cyclic cross-correlation results at *k* = 2048 are mainly used in the following for the interference spatial spectrum estimation.

#### 3.1.2. Spatial Spectrum Reconstruction of the Interference Signal based on the Sparse Estimation

The CCC result at *k* = 2048 described by Equation (8) can be rewritten as:

(9) Y=PB+Z

where:

$$Y={\left[y_{1}(2048),y_{2}(2048),\ldots ,y_{M}(2048)\right]}^{T}$$

(10) $$P=\left[\begin{array}{ccccccc} p_{1,1,1} & p_{1,2,1} & \cdots & p_{1,N_{I}{}^{1},1} & p_{2,1,1} & \cdots & p_{S,N_{I}{}^{S},1}\\ p_{1,1,2} & p_{1,2,2} & \cdots & p_{1,N_{I}{}^{1},2} & p_{2,1,2} & \cdots & p_{S,N_{I}{}^{S},2}\\ \cdots & \cdots & \cdots & \cdots & \cdots & \cdots & \cdots \\ p_{1,1,M} & p_{1,2,M} & \cdots & p_{1,N_{I}{}^{1},M} & p_{2,1,M} & \cdots & p_{S,N_{I}{}^{S},M} \end{array}\right]$$

is a $M\times N_{I}$ matrix with each column representing the steering vector of the interference signal, $N_{I}=\sum_{s=1}^{S}N_{I}{}^{s}$ is the total number of the interference signal.

$$B={\left[\begin{array}{ccccccc} B_{1,1} & B_{1,2} & \ldots & B_{1,N_{I}{}^{1}} & B_{2,1} & \ldots & B_{S,N_{I}{}^{S}} \end{array}\right]}^{T}$$

Equation (9) describes a typical linear data generating model, with Y representing the measurement data, P the data generating matrix, and B the variable to estimate. The compressed sensing technique can be used to estimate the signal DOA according to model Equation (9).

In this paper, the sparse reconstruction method proposed in literature [27] is used to estimate the DOAs of the interference. The basic idea is to solve the *l*_1_-norm minimum optimization problem as follows:

(11) $$\underset{x}{\min}\;{\left\|Ax-y\right\|}_{2}^{2}+\lambda {\left\|x\right\|}_{1}$$

where ***x*** is an M-dimensional vector comprising the amplitude of each interference signal; ${\left\|x\right\|}_{1}$ is the l1-norm of the vector; λ is the penalty coefficient; ***y*** is the measurement vector shown previously; and ***A*** is the dictionary matrix, of which each column is a steering vector corresponding to a specific DOA. For a uniform linear array, the dictionary matrix ***A*** can be formulated as follows:

(12) $$A=\left[\begin{array}{cccc} e^{j\pi \sin\left(\frac{-N_{A}}{2N_{A}}\pi \right)} & e^{j\pi \sin\left(\frac{-N_{A}+1}{2N_{A}}\pi \right)} & \cdots & e^{j\pi \sin\left(\frac{-N_{A}+2N_{A}}{2N_{A}}\pi \right)}\\ e^{j2\pi \sin\left(\frac{-N_{A}}{2N_{A}}\pi \right)} & e^{j2\pi \sin\left(\frac{-N_{A}+1}{2N_{A}}\pi \right)} & \cdots & e^{j2\pi \sin\left(\frac{-N_{A}+2N_{A}}{2N_{A}}\pi \right)}\\ \cdots & \cdots & \cdots & \cdots \\ e^{jM\pi \sin\left(\frac{-N_{A}}{2N_{A}}\pi \right)} & e^{jM\pi \sin\left(\frac{-N_{A}+1}{2N_{A}}\pi \right)} & \cdots & e^{jM\pi \sin\left(\frac{-N_{A}+2N_{A}}{2N_{A}}\pi \right)} \end{array}\right]$$

where $2N_{A}$ is the number of grids into which the azimuth space−90 ~ 90° is divided. The accuracy of the DOA estimation is related to the grid length.

The optimization problem shown in Equation (10) is to obtain an estimate of ***x*** as sparse as possible and make the mean square error lowest at the same time. Because the co-channel interference signals are widely distributed and the number of the interference signals is greater than the antenna size, Equation (10) cannot get an accurate estimate of the amplitudes for all the interference signals. However, the amplitudes as well as the DOAs of interference signals with relatively great energy can be well estimated. These are the characteristics of the LMSE criteria.

Each vector in Equation (10) is a complex vector. In the following we recast the estimation problem in Equation (10) into an equivalent one in the real-valued domain:

(13)    min A˜xRexIm−Y˜22+λ1Tus.t. xRe[m]2+xIm[m]2≤u[m], m=1,⋯M

where:

A˜=Re(A)−Im(A)Im(A)Re(A)Y˜=Re(Y)Im(Y)

Re(A) means taking the real part of matrix A and Im(A) means taking the imaginary part of matrix A.

The expression $u=\left[u[1],\cdots ,u[M]\right]$ is an auxiliary variable; xRe and xIm are the real and imaginary parts of the x.

Equation (12) can further be written by using the interior point method:

(14) minmize  tA˜[xRexIm]-Y˜22+tλ1Tu+ϕ(xRe,xIm,u)

where ϕ(xRe,xIm,u)=−∑m=1Mlog(u[m]2−xRe[m]2−xIm[m]2), t is a scalar. Equation (13) is a quadratic convex optimization problem and can be solved by the Newton method [12].

We can get x=xRe2+xim2 by solving Equation (13), then the direction of the interference signal can be determined according to the magnitude of each entity in ***x***. We denote the estimated DOA by $\theta_{r}$, $r=1,2,\cdots ,N_{R}$ with $N_{R}$ equal to the number of non-zero entities in ***x*** reconstructed. According to the RIP criteria, the relationship between $N_{R}$ and the dimension of matrix ***A***, i.e., $M\times 2N_{A}$, can be described as $M\geq cN_{R}\log(2N_{A}/N_{R})$, where *c* is very small constant.

### 3.2. Co-Channel Interference Suppression based on Spatial Feature Cognition

On getting the DOA of the interference, we use the beamforming technique to obtain the reference signal of each interference as:

(15) $$S_{ref_{r}}(n)=WS_{\mathrm{echo}}(n)\;r=1,2,\cdots ,N_{R}\;n=1,\ldots ,N$$

where $S_{\mathrm{echo}}(n)={\left[S_{1}(n),S_{2}(n),\cdots ,S_{M}(n)\right]}^{T}$ is the surveillance signal vector received by the antenna array; and W is the weight vector obtained by using the worst-case optimization beamforming algorithm [25], which can extract the reference signal of an interference with a specific DOA and can also suppress the other interferences, thus can get a good INR.

The signal extracted above can be further purified by re-generating the signal with the signal bits demodulated from $S_{ref_{r}}(n)$. We denote by ${\tilde{S}}_{ref_{r}}(n)$, $r=1,2,\cdots ,{\tilde{N}}_{R}$ the purified signal, where ${\tilde{N}}_{R}$ is the signal sample number of the re-generating signal with the incorrect demodulated bits removed.

On getting the reference signal ${\tilde{S}}_{ref_{r}}(n)$, ECA-B can be used to suppress the interference signal from a specific CCBS, i.e.,

(16) $$S_{c_{m}}=S_{m}-S_{I}(S_{I}{}^{H}S_{I}{)}^{-1}S_{I}{}^{H}S_{m}\;m=1,\cdots M$$

where $S_{m}={\left[S_{m}(0),S_{m}(1),\cdots ,S_{m}(N)\right]}^{T}$ is the surveillance signal received by the antenna element m.

The expression $S_{I}=\left[{\tilde{S}}_{I_{1}},{\tilde{S}}_{I_{2}},\cdots {\tilde{S}}_{I_{{\tilde{N}}_{R}}}\right]$ is the subspace matrix of the interference signal constructed by the reference signal; and ${\tilde{S}}_{I_{r}}$ is subspace matrix generated by the *r*-th reference signal, i.e.,

(17) $${\tilde{S}}_{I_{r}}=\left[\begin{array}{cccc} {\tilde{S}}_{ref_{r}}(0-L) & {\tilde{S}}_{ref_{r}}(0-L+1) & \cdots & {\tilde{S}}_{ref_{r}}(0-L+2L)\\ {\tilde{S}}_{ref_{r}}(1-L) & {\tilde{S}}_{ref_{r}}(2-L+1) & \cdots & {\tilde{S}}_{ref_{r}}(2-L+2L)\\ \cdots & \cdots & \cdots & \cdots \\ {\tilde{S}}_{ref_{r}}(N-L) & {\tilde{S}}_{ref_{r}}(N-L+1) & \cdots & {\tilde{S}}_{ref_{r}}(N-L+2L) \end{array}\right]$$

where *L* is the maximum delay bin. In Equation (16), each column of the interference subspace matrix is a delayed replica of the reference signal. The delay can be positive and negative, because the reference signal obtained may corresponding to a multipath signal, not the direct signal.

The worst-case optimization beamforming technique is exploited again to further suppress the interference residual, i.e.,

(18) $$S_{\mathrm{echo}\_b}(n)=WS_{c}(n)\;\;n=1,\ldots ,N$$

where $S_{c}(n)=[S_{c_{1}}(n),S_{c_{2}}(n),\cdots S_{c_{M}}{\left(n)\right]}^{T}$ is the interference residual after time domain cancellation; *W* is the beamforming weight.

After the temporal and spatial suppression, the cascaded cancellation for one loop is finished. At this step, we estimate the INR of the interference residual remaining in the surveillance signal to assess the interference level. If the INR is still high, i.e., it is larger than a predefined threshold η, then the cancellation loop is repeated. According to [10], the INR of the co-channel interference can be estimated as:

(19) $$INR\approx \frac{L_{sym}y_{\mathrm{xor}}[2048]}{L_{cp}y_{\mathrm{xor}}[0]-L_{sym}y_{\mathrm{xor}}[2048]}$$

where $L_{sym}$ is the length of an OFDM symbol and it is 2048 + 144 according to the LTE protocol; $L_{cp}$ is the length of CP in one OFDM symbol and it is 144 in LTE; and $y_{\mathrm{xor}}[k]$ is expressed as:

(20) $$y_{\mathrm{xor}}[k]=\sum_{n=1}^{N}S_{\mathrm{echo}\_b}(n)S^{*}{}_{\mathrm{echo}\_b}(n+k)$$

## 4. Simulation Analysis

**Figure 8:**
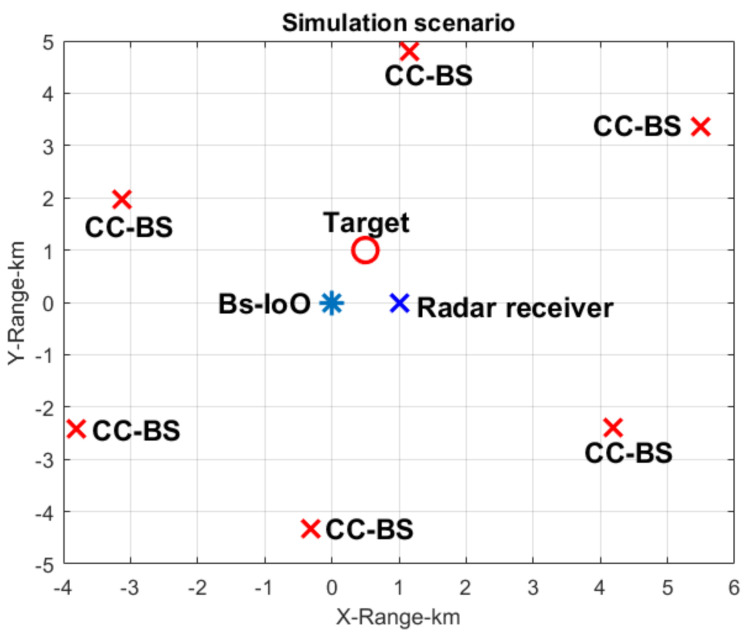
Simulation scenario.

In this section, we test the proposed method through simulations. The simulation scene is set as shown in Figure 8. It is known that in urban and suburban areas, the coverage of one LTE base station is approximately 0.3 to 1 km, so we set the distance between the BS-IoO and the radar receiver to 1 km. Six CCBSs are randomly distributed around the BS-IoO, and they are 5 km away from the BS-IoO. We simulate one target, which is 1.1 km away from the BS-IoO, and the DOA of the target is 0°. It can be seen from the simulation scene that the surveillance signal received by the radar receiver consists of the DMI from the BS-IoO and those from the CCBSs and the target echo. In this simulation, we set 15 multipath signals for the BS-IoO and 10 multipath signals originating from each CCBS. The DOA of the BS-IoO is −45° and those of the CCBSs are −30°, 20°, 70°, −66°, −20°, and 10°, respectively. The energy of the target echo is set to 70 dB smaller than the co-channel interference. Such a weak target corresponds to an unmanned aerial vehicle (UAV) of which the typical radar cross section (RCS) is only 0.01 m^2^.

**Figure 9:**
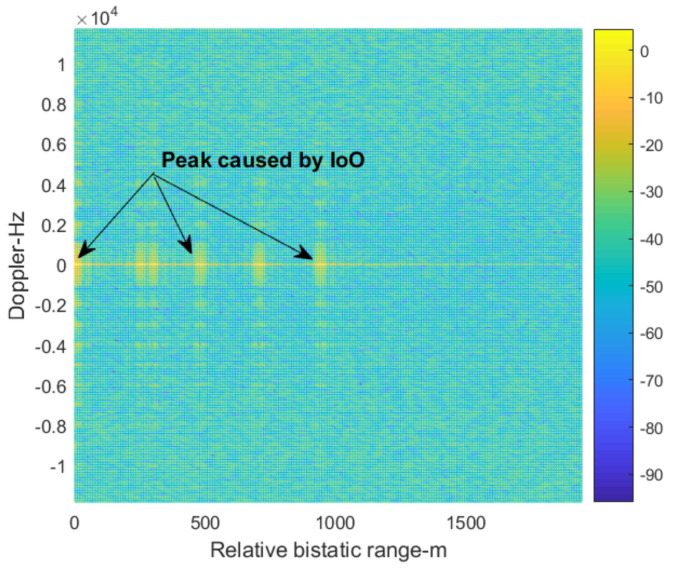
The cross-correlation results of the surveillance signal.

In the following, we set the number of the antenna array elements at 16. The spacing between two adjacent elements is half the wavelength. Because the location of the BS-IoO is known prior, we first form a beam in the direction of the BS-IoO to receive the direct signal and purify it by using the demodulation and reconstruction technique. We then use the direct signal extracted as the reference signal and cross correlate it with the surveillance signal received by one of the antenna array elements. The result is shown in Figure 9. It can be seen from Figure 9 that there are many peaks in the zero-Doppler slice. This is because of the DMI from the BS-IoO. Target echo is masked below the sidelobes of these peaks.

**Figure 10:**
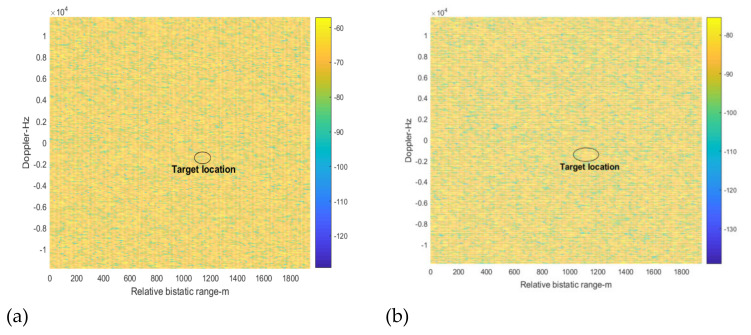
The cross-correlation results after interference suppression by the traditional method, (**a**) spatial-time (**b**) time-spatial.

In the following, we use the traditional spatial-time interference suppression method and time-spatial method to suppress the DMI and co-channel interference. The range Doppler cross-correlation result between the direct signal from the BS-IoO and the surveillance signal after interference suppression is shown in Figure 10. It can be seen that the DMI is suppressed, as there is no apparent peak in the zero-Doppler slice. However, the target still cannot be detected. This is because the co-channel interference is not suppressed adequately by the traditional method.

**Figure 11:**
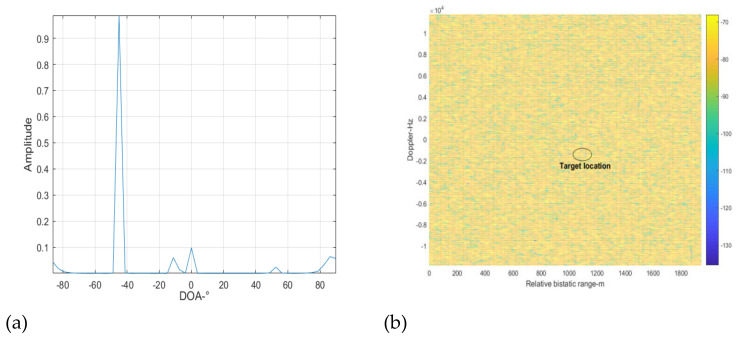
Cross correlating result at cancellation loop 3, (**a**) spatial spectrum reconstruction results, (**b**) cross-correlation results.

In the following, we use the proposed cascaded cancellation method shown in Figure 3 to suppress the interference signal. According to the processing procedure shown in Figure 3, the spatial spectrum of the surveillance signal is first constructed. The result is shown in Figure 11. It can be seen from Figure 11a that there is an apparent peak at the DOA of 45°. (It should be noted that the signal amplitude in Figure 11a is that after energy normalization.) This corresponds to the direct signal of the BS-IoO, which has the biggest SNR. The results show that the proposed method can accurately estimate the DOA of the strongest interference signal, although the number of the interference signals is far greater than that of the array elements. According to the RIP criteria used in the sparse reconstruction, the number of interference signals of which the DOAs can be accurately estimated is 4, given a 16-element antenna array and a 16×180 dimensional measurement matrix. In this simulation, we extract two peaks from Figure 11 with the largest amplitudes to estimate the DOAs of the two strongest interference signals. The other peaks may correspond to noise and may not indicate the DOA of a specific interference signal. The estimated DOAs of the two strongest interference signals are −45 and 1 degrees, respectively. We then form two beams to obtain the two interference signals. We find from the demodulation results that the signal at 1° is a multipath version of the signal at −45°. We then use the ECA-B algorithm to suppress the interference signal with the purified signal at −45 degrees as the reference signal. After that, the cross-correlation result is shown in Figure 11b. It can be seen that the peaks caused by the DMI from the BS-IoO have vanished. This shows that the DMI was cancelled adequately. However, the target is still not detectable. This is because of the presence of the co-channel interference. The INR estimated is larger than the predefined threshold, i.e., 5 dB in this simulation. The above cancellation loop is repeated in the following.

**Figure 12:**
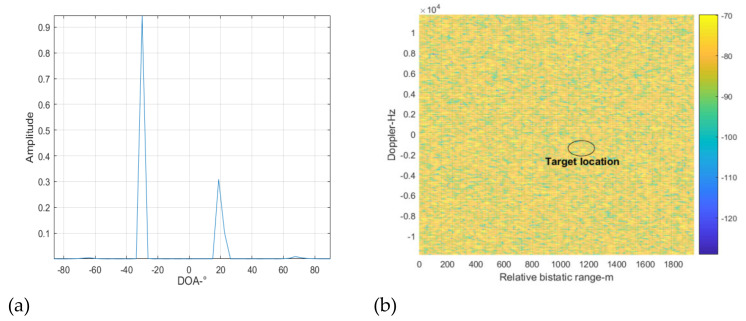
Cross-correlating result at cancellation loop 2, (**a**) spatial spectrum reconstruction results, (**b**) cross-correlation results.

Figure 12 shows the spatial spectrum reconstruction result at cancellation loop 2. Because the DMI from the BS-IoO was cancelled at cancellation loop 1 above, the interference signals indicated by the peaks in Figure 12a correspond to the CCBSs. Similarly, we extract two peaks from Figure 12a with the largest amplitudes, from which the DOAs of the two corresponding interference signals are estimated as −30 and 20 degrees. Next, we use the beamforming and purification techniques to get the clean versions of the reference signals at −30 and 20 degrees. ECA-B is then performed with the reference signals obtained to suppress the interferences. The cross-correlation result at this step is shown in Figure 12b. It can be seen that the target is still not detectable, because the interference residual is still large and the INR of the interference residual estimated is still larger than 5 dB. Therefore, the cancellation loop needs to repeat again.

**Figure 13:**
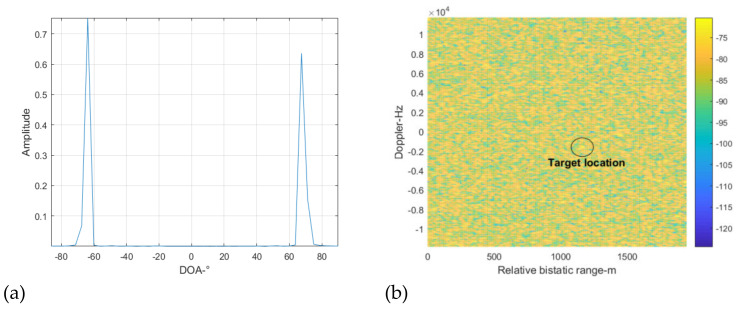
Cross-correlating result at cancellation loop 3, (**a**) spatial spectrum reconstruction results, (**b**) cross-correlation results.

Figure 13 shows the spatial spectrum reconstruction result at cancellation loop 3. We extract the two apparent peaks in the figure to estimate the DOAs of the two corresponding interference signals, which are −67 and 69 degrees, respectively. Next, the beamforming and purifying techniques are used to get the clean versions of the interference signals at the two DOAs, and ECA-B is performed to suppress the corresponding interference. The cross-correlation result at this step is shown in Figure 13b. The target is still not detectable and the INR of the interference residual estimated is larger than 5 dB. The cancellation loop needs to be repeated once more.

**Figure 14:**
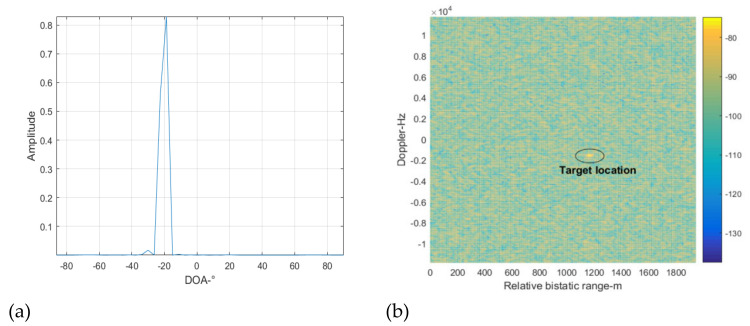
Cross-correlating result at cancellation loop 4, (**a**) spatial spectrum reconstruction results, (**b**) cross-correlation results.

The spatial spectrum reconstruction result at cancellation loop 4 for the remaining signal is shown in Figure 14. It can be seen that there is one apparent peak. We extract this peak to estimate the DOA of the corresponding interference signal, which is −19 degrees. Next the beamforming and purifying techniques are used to get the clean versions of the interference signals at this DOA, and ECA-B is performed to suppress the corresponding interference. The cross-correlation result at this step is shown in Figure 14b. It can be seen that the target is clearly seen in the range Doppler surface. The INR of the interference residual estimated is smaller than the predefined threshold, and the cancellation loop terminates.

In the following, we investigate the influence of the antenna array size on the suppression performance of the proposed method. We set one BS-IoO and six CCBSs. Fifteen DMI from the BS-IoO are simulated and five multipath signals for each CCBS are simulated. The direct signal to noise ratio of the BS-IoO at the radar receiver is set to 50 dB. The SNRs of the other interference signals are randomly simulated 12 dB smaller than the BS-IoO direct signal. One target is simulated with an SNR of −25 dB.

**Figure 15:**
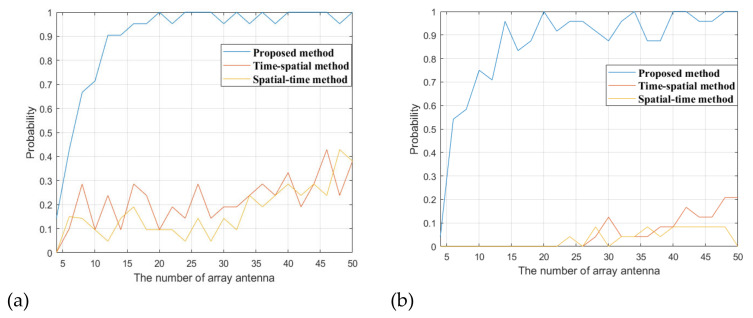
Comparison of interference suppression performance under different number of antenna arrays, (**a**) interference from the sidelobe, (**b**) interference from the main lobe.

We vary the antenna array element number from 4 to 16. For each element number, 100 Monte Carlo simulations are conducted and the detection probability of the target is counted. If the energy of the target is 5 dB above the interference floor after the final cancellation loop, then we declare the detection of the target. The detection probability when the traditional methods are used to suppress the interference is also calculated. The simulation results are shown in Figure 15.

Figure 15a shows the results when all the interference signals are from the sidelobe of the surveillance beam. It can be seen that the proposed method can get much better target detection probability than the traditional methods for all the array element numbers. It can get a detection probability of above 0.9 when the antenna array element number exceeds 12. However, the traditional methods can only get a detection probability of less than 0.5 for most of the cases. This shows that the proposed method can adequately cancel the DMI and co-channel interference from the BS-IoO and CCBSs, whereas the cancellation of those interference signals is not sufficient when using the traditional methods.

Figure 15b shows the results when some of the co-channel interference signals are from the main lobe of the surveillance beam. It can be seen that in this circumstance the detection probability obtained by all the methods degrades. However, the proposed method still has a detection probability of above 0.8 when the antenna array element number exceeds 12. The detection probability obtained by the traditional methods degrades significantly, and it is smaller than 0.2 for all the array element number cases.

**Figure 16:**
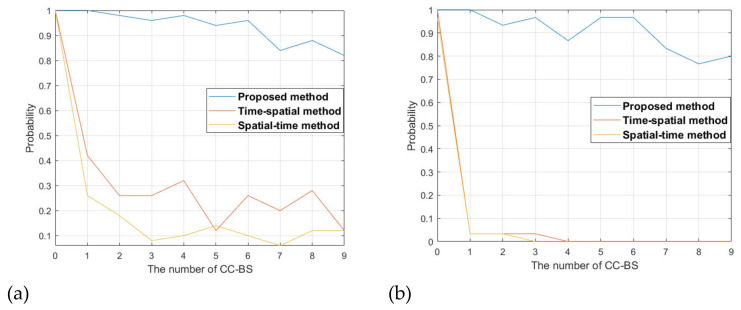
Comparison of interference suppression performance under different number of CCBSs, (**a**) interference from the sidelobe, (**b**) interference from the main lobe.

In the following, we fix the antenna array element number to 16 and vary the number of CCBSs to see the detection performance of the target obtained by the proposed and traditional methods. The number of the CCBSs varies from 0 to 9. For each CCBS number, 100 Monte Carlo simulations are conducted. The simulations results are shown in Figure 16.

Figure 16a shows the results when all the interference signals are from the sidelobe of the surveillance beam. It can be seen that when the number of the CCBSs is zero, i.e., there is only the DMI from the BS-IoO and no co-channel interference, the proposed and traditional methods get a similar detection probability. As the CCBS number increases, the detection probability from the proposed and traditional methods decreases. This is because the interference signals become severe when the CCBS number increases. However, the detection probability degrades significantly for the traditional methods and it is smaller than 0.3 when the number of CCBSs exceeds 1. The proposed method still gets good detection probability, above 0.8 for all the CCBS number cases.

Figure 16b shows the results when some of the interference signals are from the main lobe of the surveillance beam. It can be seen that the traditional methods degrade severely when there exists CCBSs, and for most cases the target is not detectable because the SNR of the target is smaller than 5 dB. This shows that the traditional methods have very limited ability to handle the case when the interference signals are from the main lobe of the surveillance beam. The proposed method can still get good detection performance and the detection probability is above 0.8 for most of the CCBSs cases. The degradation compared with the case where the interference signals are from the sidelobe is not apparent.

## 5. Conclusions

The paper discussed the DMI and co-channel interference suppression problem in LTE based passive radar. We proposed a cascade cancellation method that can effectively suppress the DMI and co-channel interference with a small sized antenna array. A DOA estimation method based on the cyclostationary characteristics of the OFDM signal and the sparse reconstruction technique was also proposed. It can obtain good DOA estimation of strong interference when the number of interference signals far exceeds that of the array elements. As can be seen in our simulation shown in Figure 15, 45 interferences exist (15 DMI from the BS-IoO and 30 DMI from thesix CCBSs), but the proposed method could still obtain effective interference suppression and achieve a detection probability of above 0.9 by only using a 12-element antenna array. The detection performance can be further refined by using tracking filter methods, such as the Kalman tracking filter, which can eliminate the false alarms caused by the residual interference.

## Data Availability

The study did not report any data.
